# Age-related deficit accumulation and the risk of late-life dementia

**DOI:** 10.1186/s13195-014-0054-5

**Published:** 2014-09-18

**Authors:** Xiaowei Song, Arnold Mitnitski, Kenneth Rockwood

**Affiliations:** 1 Department of Medicine, Dalhousie University, Halifax, NS, Canada; 2 Centre for Health Care of Elderly, Division of Geriatric Medicine QEII Health Sciences Centre, Capital District Health Authority, Halifax, Canada; 3 Departments of Mathematics and Computer Science, Dalhousie University, Halifax, Canada

## Abstract

**Introduction:**

Many age-related health problems have been associated with dementia, leading to the hypothesis that late-life dementia may be determined less by specific risk factors, and more by the operation of multiple health deficits in the aggregate. Our study addressed (a) how the predictive value of dementia risk varies by the number of deficits considered and (b) how traditional (for example. vascular risks) and nontraditional risk factors (for example, foot problems, nasal congestion) compare in their predictive effects.

**Methods:**

Older adults in the Canadian Study of Health and Aging who were cognitively healthy at baseline were analyzed (men, 2,902; women, 4,337). Over a 10-year period, 44.8% of men and 33.4% of women died; 7.4% of men and 9.1% of women without baseline cognitive impairment developed dementia. Self-rated health problems, including, but not restricted to, dementia risk factors, were coded as deficit present/absent. Different numbers of randomly selected variables were used to calculate various iterations of the index (that is, the proportion of deficits present in an individual. Risks for 10-year mortality and dementia outcomes were evaluated separately for men and women by using logistic regression, adjusted for age. The prediction accuracy was evaluated by using C-statistics.

**Results:**

Age-adjusted odds ratios per additional deficit were 1.22 (95% confidence interval (CI), 1.18 to 1.26) in men and 1.14 (1.11 to 1.16) in women in relation to death, and 1.18 (1.12 to 1.25) in men and 1.08 (1.04 to 1.11) in women in relation to dementia. The predictive value increased with the number *(n)* of deficits considered, regardless of whether they were known dementia risks, and stabilized at *n* > 25. The all-factor index best predicted dementia (C-statistics, 0.67 ± 0.03).

**Conclusions:**

The variety of items associated with dementias suggests that some part of the risk might relate more to aberrant repair processes, than to specifically toxic results. The epidemiology of late-life illness might best consider overall health status.

## 1 Introduction

A growing number of factors are associated with dementia risk. Reports just from 2014 reify vascular risk factors (in the degree of intracranial artery stenosis) [1], implicate impaired sleep [2], and raise questions about gynecologic surgery [3], and pesticides [4]. Even recognizing that seemingly disparate factors might share common mechanisms, it remains unclear what to make of the number and diversity of risk factors. Their very disparity might hold a clue. Inasmuch as the risk factors appear to have so little in common, their collective role might simply be to induce aberrant repair processes [5].

Despite these many new exposures associated with dementia, age remains the single biggest risk factor. Likewise, it is also the biggest risk factor for death, and this may help us understand how to interpret the growing list of dementia risk factors. The risk of death increases exponentially with age, but not everyone of the same age has the same risk of dying. People at an increased risk of death compared with others of the same age are said to be frail [6]; this greater risk typically obtains for other adverse outcomes, too, including, institutionalization, health service use, and worse health.

How best to make operative the concept of frailty is disputed, but as detailed later, both well-established methods (that is, the frailty syndrome/phenotype and a frailty index (FI) based on the accumulation of health deficits) have been linked to late-life cognitive impairment. The FI counts how many deficits a person has (broadly defined by biological and clinical characteristics); it can be calculated simply as the ratio of the deficits present in a person to the total number of deficits considered in a given study setting [7]. A possibility linking age-related accumulation of health deficits and dementia is that the latter represents the failure of a high order, integrative function (for example, cognition) in a system that is close to failure (for example, in people who are frail) [8]. By that line of reasoning, late-life dementia can be understood with less attention to specific risk factors, and more to how these risk factors operate in the aggregate [9]. In other words, just as the higher risk of death in older adults reflects their frailty more than it does their age, so too might the increased risk of dementia arise in relation to their general health status, which can be represented by the number of health deficits that they have accumulated.

Some of this is not new, in that, for example, dementia risk is known to be related to the combined effect of many factors [10]-[14]. Likewise, several reviews suggest associations between frailty and cognitive decline [15],[16], especially when death is modeled as a competing risk [9]. As Barnes and Lee [9] pointed out, the most widely used approach to dementia prediction uses weighted scales that combine a small number of selected risk factors, each of which is significantly associated with dementia [9]. Even so, only moderate accuracy results [9],[10],[17]. Notably, many such scales involve age and genetic risk factors (for example, ApoE4) that are hardly modifiable [9],[17]. Approaches to pick the best possible predictors, including the best number, and their optimal weights are difficult: empirically, multiple combinations of different variables can have equivalent value in predicting outcomes.

Such considerations also inform our approach, but with a different take that, in our view, has consequences for both dementia epidemiology and potentially for management. Instead of picking out factors that only individually are statistically significant, and then seeing whether they survive multivariable modeling, we were struck by the fact that different subsets of variables can give comparable predictions. This suggests both considerable diversity in individual health and multiple dependencies among health measures [8]. In other words, seemingly insignificant factors, as judged by statistical considerations alone, can still impact the system and modify risk.

When these seemingly insignificant factors are combined, they may help reveal the state of the system [7],[8]. In this regard, we reported that poor general health, as manifest by the presence of multiple health deficits, increases dementia risk [18]. Note that this was shown to be the case for health deficits that otherwise were not known to be directly linked to dementia. Note too that these factors worked in combination; indeed, in contrast to the convention of including in the multivariable risk model only items that were individually significantly associated with dementia, we proposed an index made up solely of nontraditional risk factors (that is, the frailty index of nontraditional risk factors), most of which were not individually associated with dementia risk [18]. That approach (of including all risks, without regard to their individual statistical significance) borrows from signal-detection methods long used in information theory [19]. Recently, again by using this approach, our group has replicated the finding that nontraditional risk factors combine to predict cognitive decline [20].

To understand better how frailty and age influence dementia risk, we now consider (1) how traditional (including vascular risk factors and cognition-related measures) and nontraditional risk factors (including measures of health that are not considered as dementia risks) compare in their predictive ability, and (2) how the predictive value of dementia risk might vary by the number of deficits considered. We addressed these questions by reevaluating the Canadian Study of Health and Aging (CSHA), an established cohort with 10-year dementia follow-up. Multiple indices were constructed by using various numbers of randomly selected deficits that either have or have not been associated with dementia risks. To isolate dementia from death, these outcomes were assessed separately. Given the likely gender differences in dementia and mortality in relation to deficit accumulation [8],[21],[22], we evaluated the risks separately for men and women.

## 2 Methods

### 2.1 Participants

**Figure 1:**
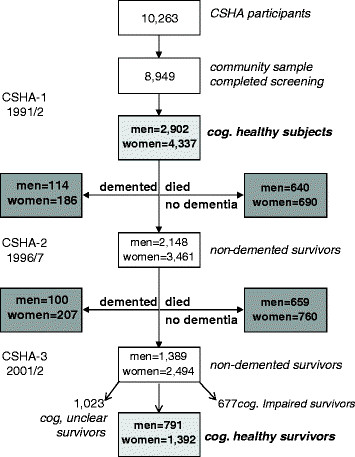
**Flow diagram showing the sample.** The CSHA assembled a representative cohort of 10,263 participants aged 65 years and older in 1991/1992 (CSHA-1) in all Canadian provinces, with follow-ups occurring in 1996/1997 (CSHA-2) and 2001/2002 (CSHA-3) [CSHA 2000]. At baseline, community-dwelling older adults were screened, and self-reported health evaluation data were available in 8,940 participants who completed the baseline survey. Global cognitive assessment was made with use of the 100-point Modified Mini-Mental State Examination (3MS) [Teng EL, Chui HC. **The Modified Mini-Mental State (3MS) examination.***J Clin Psychiatry* 1987, **48**; 314–318.]. People who had 3MS total scores ≤78 were invited to have a detailed clinical cognitive examination, at which time, a clinical diagnosis was made. Cognitive status of all participants was assessed at baseline and at both 5-year and 10-year follow-ups. Subjects who were assessed as cognitively intact at baseline, based on negative screening (3MS >78) and/or a negative clinical diagnosis (2,902 men and 4,337 women) were further analyzed.

This is a secondary analysis of data from the Canadian Study of Health and Aging (CSHA). The CSHA was a well-characterized, nationwide, multicenter, dementia epidemiology study that assembled a representative cohort of 10,263 participants aged 65 years and older in 1991/1992 (CSHA-1) in all Canadian provinces (Figure 1) [18]. Participants living in the community (*n* = 8949) were first interviewed in their homes to record general health information and to screen for possible dementia. The community interview covered general health, disability, social circumstances, and the presence of chronic health problems. In the interview, the Modified Mini-Mental State examination (3MS) was used to screen for cognitive impairment (for example, 3MS ≤78). Participants who screened positive for cognitive impairment, and a random subsample of people who screened negative, were asked to attend a clinical assessment. Two 5-year follow-ups occurred in 1996/1997 (CSHA-2) and 2001/2002 (CSHA-3), at which time all the surviving participants who had previously had a clinical examination were invited to be reexamined [18]. For study subjects who had died before one of the follow-up studies, a relative was interviewed to collect information on cognitive and physical health during the last months of the person’s life.

In each wave, the final diagnosis of dementia was made at a consensus interview, in which a combination of medical and neuropsychological assessments administered in the patient’s home or at a clinic were considered. This was done, with the nurse, after the physician and neuropsychologist had independently made preliminary diagnoses. At the consensus diagnosis, the panel classified people as demented, cognitively impaired but not demented (CIND), or as cognitively normal. The same diagnostic criteria were used at follow-ups for comparability with previous diagnoses, and rediagnosed according to new DSM-IV criteria that had been developed only after the study began.

For this secondary analysis of the CSHA community sample from CSHA-1 to CSHA-3, subjects who were cognitively healthy at baseline (2,902 men and 4,337 women), based on negative screening and/or a negative clinical diagnosis, were further analyzed (Figure 1). Over the 10-year follow-up, 214 men (7.4%) and 393 women (9.1%) developed dementias of various subtypes (including 416 with Alzheimer disease and 191 with vascular, mixed, Parkinson, frontotemporal, Lewy body, and other dementias); another 1,299 men (44.8%) and 1,450 women (33.4%) died; 791 men and 1,392 women remained cognitively healthy (Figure 1; Table 1).

**Table 1:** Variables used to construct the indices by sex, in relation to mortality and dementia outcomes

**Variable**	**Men**						**Women**					
	**Problem absent (value = 0)**			**Problem present (value = 1)**			**Problem absent (value = 0)**			**Problem present (value = 1)**		
	***N***	**Die (%)**	**Dement (%)**	***N***	**Die (%)**	**Dement (%)**	***N***	**Die (%)**	**Dement (%)**	***N***	**Die (%)**	**Dement (%)**
How is your health these days? (1 = not too good to very poor; 0 = very good or pretty good)	2,819	44.1	7.4	75	70.7	4.0	4,222	32.7	9.2	107	61.7	4.7
How good is your eyesight? (1 = poor or unable to see; 0 = excellent, good, or fair)	2,776	43.9	7.3	125	64.0	8.0	3,996	31.7	8.8	339	53.1	11.8
How good is your hearing? (1 = poor or unable to hear; 0 = excellent, good, or fair)	2,750	44.4	7.1	152	51.3	12.5	4,153	32.5	9.1	183	54.6	9.3
Dentures fit to your satisfaction? (1 = no; 0 = yes)	2,551	44.5	7.3	345	46.7	8.1	3,741	32.4	9.2	581	40.1	8.1
Arthritis or rheumatism? (1 = yes; 0 = no)	1,543	44.0	7.3	1,355	45.5	7.5	1,591	31.2	9.3	2739	34.7	8.9
Eye trouble? (1 = yes; 0 = no)	2,259	41.4	7.4	640	56.6	7.2	2,861	29.3	8.1	1470	41.4	10.8
Ear trouble? (1 = yes; 0 = no)	2,021	41.8	6.7	880	51.7	8.9	3,245	31.0	8.7	1087	40.8	10.0
Trouble with your stomach? (1 = yes; 0 = no)	2,313	43.6	7.3	587	49.4	7.5	3,091	31.7	9.5	1241	37.8	8.0
Kidney trouble? (1 = yes; 0 = no)	2,633	43.4	7.7	266	58.3	4.5	3,823	32.0	9.3	506	44.5	7.7
Lose control of your bladder? (1 = yes; 0 = no)	2,669	43.5	7.5	226	60.2	6.2	3,524	31.6	9.3	804	41.4	8.3
Lose control of your bowels? (1 = yes; 0 = no)	2,810	44.3	7.3	89	57.3	9.0	4,114	32.8	9.1	216	44.9	7.9
Trouble with your feet or ankles? (1 = yes; 0 = no)	2,204	41.2	7.3	694	56.1	7.6	2,714	30.4	8.3	1613	38.7	10.4
Nose stuffed up or sneezing? (1 = yes; 0 = no)	2,453	43.6	7.1	446	51.3	9.0	3,607	32.7	8.8	724	37.2	10.4
Any fractures? (1 = yes; 0 = no)	2,787	44.6	7.4	109	50.5	7.3	4,027	32.9	9.1	299	40.5	9.0
Chest problems? (1 = yes; 0 = no)	2,345	41.2	7.8	555	59.8	5.8	3,660	31.4	9.5	671	44.6	6.4
Have you had a cough? (1 = yes; 0 = no)	2,541	43.0	7.6	359	57.1	5.8	3,798	32.8	9.1	534	38.2	8.8
Skin problems? (1 = yes; 0 = no)	2,356	44.6	7.1	539	45.8	8.3	3,541	32.8	8.9	790	36.1	9.9
Dental problems? (1 = yes; 0 = no)	2,335	43.9	7.3	555	49.0	7.7	3,483	33.1	9.2	839	34.8	8.7
Have you had any other problem? (1 = yes; 0 = no)	2,241	43.9	7.1	636	48.3	8.3	3,290	33.0	9.2	1025	34.9	8.3
High blood pressure? (1 = yes; 0 = no)	2,090	42.9	7.6	803	49.7	6.6	2,632	31.2	9.3	1694	37.0	8.7
Heart and circulation problems? (1 = yes; 0 = no)	2,034	39.1	7.5	864	58.0	7.1	3,008	28.4	9.4	1319	45.1	8.3
Stroke or effects of stroke? (1 = yes; 0 = no)	2,757	43.9	7.3	142	63.4	7.7	4,174	32.8	8.9	152	50.7	12.5
Diabetes? (1 = yes; 0 = no)	2,598	43.2	7.4	301	58.1	7.6	3,930	31.9	8.1	395	47.8	9.2
Do you live here alone? (1 = yes; 0 = no)	2,393	43.0	7.3	509	52.8	7.9	2,245	28.8	8.1	2092	38.4	10.1
Can you eat? (1 = can’t do at all or with some help; 0 = without any help)	2,890	44.6	7.4	11	90.9	0.0	4,317	33.3	9.0	18	72.2	16.7
Can you dress and undress yourself? (1 = can’t do at all or with some help; 0 = without any help)	2,848	44.1	7.3	52	78.8	13.5	4,284	33.1	9.1	52	61.5	3.8
Can you take care of your appearance? (1 = can’t do at all or with some help; 0 = without any help)	2,884	44.6	7.3	16	75.0	18.8	4,283	33.1	9.0	51	58.8	17.6
Can you walk? (1 = can’t do at all or with some help; 0 = without any help)	2,827	43.7	7.4	74	85.1	6.8	4,080	31.3	8.9	255	67.5	11.4
Can you get in and out of bed? (1 = can’t do at all or with some help; 0 = without any help)	2,879	44.6	7.4	22	72.7	9.1	4,298	33.2	9.0	37	54.1	18.9
Can you take a bath or shower? (1 = can’t do at all or with some help; 0 = without any help)	2,744	43.0	7.4	157	76.4	6.4	3,798	30.4	8.7	537	54.9	11.9
Can you go to the bathroom? (1 = can’t do at all or with some help; 0 = without any help)	2,878	44.6	7.3	21	76.2	14.3	4,287	33.3	9.1	48	43.8	8.3
Can you use the telephone? (1 = can’t do at all or with some help; 0 = without any help)	2,802	44.2	7.2	100	60.0	13.0	4,197	32.7	9.0	138	56.5	10.9
Can you get to place out of walking distance? (1 = can’t do at all or with some help; 0 = without any help)	2,795	43.4	7.3	107	79.4	9.3	3,787	29.7	8.8	546	59.2	10.8
Can you go shopping? (1 = can’t do at all or with some help; 0 = without help)	2,718	42.7	7.2	182	75.8	9.3	3,543	28.0	8.4	790	57.8	12.2
Can you prepare your own meals? (1 = can’t do at all or with some help; 0 = without any help)	2,677	43.2	7.2	223	63.7	8.5	4,063	31.4	8.7	269	63.2	14.9
Can you do your housework? (1 = can’t do at all or with some help; 0 = without any help)	2,370	39.9	7.0	527	66.6	8.9	2,868	25.3	7.7	1463	49.4	11.7
Can you take your own medicine? (1 = can’t do at all or with some help; 0 = without any help)	2,842	44.2	7.2	54	74.1	14.8	4,243	32.7	9.0	85	69.4	10.6
Can you handle your own money? (1 = can’t do at all or with some help; 0 = without any help)	2,830	44.1	7.3	72	72.2	8.3	4,153	32.3	8.8	180	60.0	15.0
Have you been feeling tired? (1 = yes; 0 = no)	2,546	42.3	7.6	353	62.0	5.9	3,502	30.9	8.8	828	44.2	10.4
How is language ability? (1 = with difficulties; 0 = fluent)	2,835	44.6	7.4	64	50.0	4.7	4,287	33.5	9.1	48	29.2	8.3
Trouble with your nerves? (1 = yes; 0 = no)	2,565	44.3	7.1	335	48.7	9.6	3,391	32.8	8.9	933	36.0	9.5
Troubles prevent normal activities? (1 = a little or a great deal; 0 = not at all)	1,500	34.7	7.5	1,398	55.4	7.2	2,074	24.7	9.1	2255	41.4	9.1

### 2.2 Variables and Frailty Index construction

The FI approach based on deficit accumulation is detailed elsewhere [7],[8]. In brief, the FI evaluates the extent to which deficits are accumulated in a given person, which is quantified as the proportion of the deficits present in this person (that is the number of deficits present divided by the total number of potential deficits that were considered). This leads to an index score ranging theoretically between the possible best value of 0 (no deficits present) and the possible worst value of 1 (all deficits present, although an empiric limit of 0.7 to the FI has been demonstrated, beyond which further accumulation leads to death, so that this value is not exceeded [8]).

Here, the FI made use of self-reported health-deficit evaluations from the CSHA-1 community-sample interview that met the criteria of being health-deficit measures (that is, being biologically meaningful in representing several organ systems, accumulating with age, and not becoming too prevalent at some younger age, with >1% prevalence and <5% missing data). The FI acknowledges that various health problems can be multiply dependent and interrelated; in biological systems, this remains the case whether they are found to be statistically independent, or otherwise. Even with such overlap, the FI works by allowing small pieces of information to contribute (literally to add up) to quantify the overall state of health of an individual [6]-[8],[18],[19],[22]. In this dataset, the process resulted in a set of 42 potential deficit measures, including diseases, symptoms, signs, disabilities, and lifestyle/environmental factors (Table 1). Four of these variables (high blood pressure, heart/circulation problems, stroke history/effect, and diabetes) were recognized vascular risk factors, whereas another 19 variables (for example, poor health attitude, problems with stomach, kidney, eye, ear, teeth, or skin) were not commonly recognized as cognitive risk factors (nontraditional risk factors) [18]. The set also included 19 variables on items (such as disabilities) that are commonly used in assessing deficit accumulation, and that sometimes are proposed as dementia risk factors, but that might equally represent early disease expression [8].

Each variable was coded to a binary value 0 or 1, representing that a problem is present (“1”) or absent (“0”) [7]. The three-level variables (*n* = 8; for example, “Can you walk?”) were each dichotomized with “1” representing “cannot” or “with help”; “0” representing “yes”. Similarly, the five-level variables (*n* = 3; for example, “how is your health these days?”) were each dichotomized with “1” representing “not too good,” “poor”, or “very poor”; and “0” representing “pretty good” or “very good” (Table 1).

An index score was created for each individual simply as the sum of all deficits present divided by the total number of variable considered. For example, if someone had heart disease, stomach ache, troubles with eyes and hearing, and needed help with walking, of a total of 42 potential deficits considered, then this person’s FI score would be (5 of 42 = 0.12). The index score can then be used to assess the combined vulnerability of the deficits to adverse outcomes (for example, death and dementia) [8],[18]. Here, to test objective 1, we first created three risk-factor indices, by respectively considering each of the vascular factors (*n* = 4), nontraditional factors (*n* = 19), and then with all health deficits combined in an FI (*n* = 42). To test objective 2, indices were generated by using different numbers of randomly selected variables *n* (*n =* 1, 5, 10…40), regardless of whether they were dementia risks.

### 2.3 Outcomes

The primary outcomes were 10-year dementia and mortality, evaluated by sex. In the CSHA, as elaborated elsewhere, dementia severity and subtypes were diagnosed after a detailed cognitive examination using standard criteria [18]. Decedent data were obtained from the Registrar of Vital Statistics in each province, in addition to interviews of spouses or next of kin of the study participants who had died, verified by Statistics Canada [18]. Subjects who were known to have developed dementia and later died were counted only for dementia outcome, whereas death outcome contained those who died without dementia, as well as those for whom cognitive status, but not vital status, was unknown (due to lack of 5-year cognitive-assessment data).

### 2.4 Statistical analysis

Data were analyzed separately by sex. Group differences in demographics were examined by using ANOVA for continuous (for example, age, year of schooling) and χ^2^ test for categoric variables (for example, marital status). χ^2^ tests were also used to compare each deficit in relation to the outcomes. Pair-wise differences were examined by using the Tukey multiple comparison. Age-specific distributions of the indices were estimated as the mean values with 5-year aggregation of age from 65 years. Relations between pairs of indices were examined by using correlation and regression analyses. Rates of death and dementia were calculated for the tertiles of the sample with the lowest, medium, and the highest index scores of the vascular, nontraditional, and all (42) risk-factor risk indices.

To evaluate the risks for the 10-year death and dementia outcomes, a multivariable logistic regression model was used to calculate the odds ratios (ORs) and 95% confidence intervals (CIs) of death versus survival and of dementia versus healthy cognition in relation to the index constructed by using different numbers of deficit variables in men and in women, adjusted for age. In doing so, values of the index were presented by using the number of the deficit count (for example, multiplied the index by 42 for the all-factor measure), to evaluate the change in risk seen with each added deficit. Likewise, how the predictive value of dementia risk might vary by the number of deficits was evaluated with the change in the Wald statistic. The Wald test determines the degree of significance of any explanatory variable in a multivariable model. Given that age is strongly associated with all late-life outcomes, the change in the Wald statistic in relation to age after introduction of the various index variables is another way to understand their impact. In addition, to evaluate the predictive value for 10-year death and dementia, the receiver operating characteristics (ROCs or C-statistics) with bootstrapping and assessed by using the areas under the curves (AUCs). Repeated sampling was applied to random variable selection (1,000 times for each number of deficit variables under consideration), to cover various possibilities of variables inclusion.

The majority of men (2,826 or 97.4%) and women (4,254 or 98.1%) in the sample had no missing values in any of the 42 variables. The maximum number of missing cases was ≤0.6%, which was found in one variable. Missing values were handled by using multiple imputations, and sensitivity analysis showed no significant difference in the variables (*P* > 0.05). All analyses were performed by using SPSS Statistics version 20 and codes developed by using Mathlab R2013a. Statistical significance level was set at *P* = 0.05.

### 2.5 Standard protocol approvals, registrations, and patient consents

Data collection was approved by the CSHA ethics review process, with informed consent provided by all participants. Approval for the secondary analyses came from the Research Ethics Committee of the Capital District Health Authority, Halifax, Nova Scotia, Canada.

## 3 Results

Overall, cognitively healthy older adults (men or women) who survived 10 years were at baseline relatively younger, better educated, and had lower levels of frailty, compared with those who became cognitively impaired or demented, or who died (Table 2). Cognitively healthy survivors were also more likely to have been married. Although those who died were the least healthy at baseline, the baseline profiles of people who died or developed dementias were otherwise similar (Table 2). In each group, women were older on average and less likely to have a living spouse compared with men (Table 2). Each deficit (except language inability in women) was individually associated with an increased risk of death, whereas only some deficits (27 of 42 in men; 25 of 42 in women) were individually associated with dementia (Table 1).

**Table 2:** Baseline characteristics of the sample, by outcomes at 10 years

		**Cognitive healthy survivors**	**Cognitive-impaired/unknown survivors**	**Demented**	**Deceased**
Men (*n* = 2,902)					
	Number	791	598	214	1,299
	Age (years)	70.0 ± 4.4	72.4 ± 5.7	75.8 ± 6.0	76.0 ± 6.4
	Education (years)	12.0 ± 4.1	10.0 ± 4.3	10.7 ± 3.9	10.4 ± 3.9
	<9-year school (%)	20.8	40.3	32.9	34.1
	Being married (%)	75.0	69.7	69.2	68.0
	3MS total (per 100)	92.4 ± 5.1	88.6 ± 5.6	87.9 ± 5.6	88.7 ± 5.6
	Frailty Index (of 42)	0.09 ± 0.06	0.12 ± 0.08	0.14 ± 0.09	0.15 ± 0.09
Women (*n* = 4,337)					
	Number	1,392	1,102	393	1,450
	Age (years)	71.3 ± 4.8	75.0 ± 6.1	78.7 ± 6.0	78.3 ± 6.9
	Education (years)	11.0 ± 3.3	10.2 ± 3.4	10.5 ± 3.2	10.5 ± 3.3
	<9-year school (%)	22.7	32.4	27.1	27.0
	Being married (%)	50.8	42.3	33.1	32.8
	3MSE total (of 100)	92.6 ± 4.9	89.0 ± 5.6	87.6 ± 6.0	88.9 ± 5.8
	Frailty Index (of 42)	0.13 ± 0.08	0.16 ± 0.09	0.18 ± 0.10	0.20 ± 0.10

**Figure 2:**
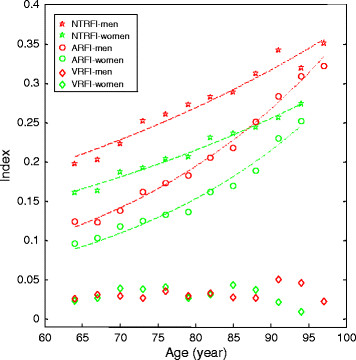
**Risk-factor indices in relation to age.** The indices containing vascular risk factors (VRFIs; *n* = 4), nontraditional risk factors (NTRFIs; *n* = 19), and all risk factors (ARFIs; *n* = 42) are presented as a function of age in men (green) and in women (red). Symbols represent the observational data as means for 3-year age groups; lines represent curve fitting. The nontraditional and all factor indices increased exponentially with age (for example, R^2^ = 0.981 in men and R^2^ = 0.939 in women for the NTRFI; R^2^ = 0.982 in men and R^2^ = 0.987 in women for the ARFI; *P* < 0.001 in each case).

**Figure 3:**
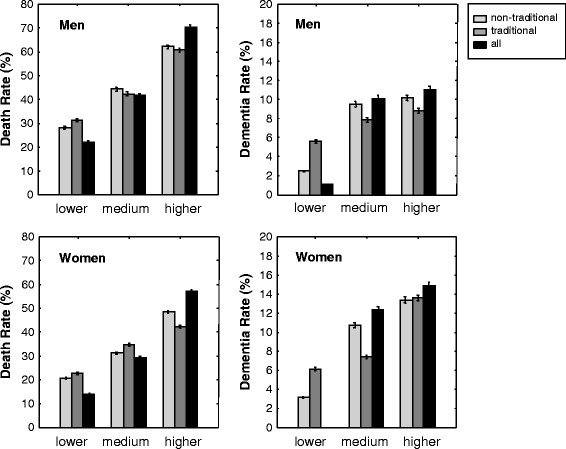
**Ten-year death (left panels) and dementia (right panels) rates in men (upper panels) and women (lower panels) as a function of deficit accumulation for the 19-item nontraditional risk-factor index (NTRFI), the four-item vascular risk-factor index (VRFI), and the 42-item all-risk-factor index (ARFI).** Data represent the mean and the variance of the population for the tertiles with the lowest (open bars), medium (light-grey bars), and the highest (dark-grey bars) index scores.

When the deficits were combined, both the frailty (all-factor) index and the nontraditional risk-factor index increased with age; this was not the case for the vascular risk-factor index (Figure 2). Adjusted for age, the odds ratios per additional deficit increased for both death and dementia in both women and men, albeit a greater odds for each adverse outcome per FI increment in men (Table 3). Regarding objective 1 (the nature of the risk factors), in each risk-factor index, subjects in the tertile with the highest index scores had the highest mortality and dementia rates, whereas those with the lowest index scores had the lowest, showing that seldom did a person with an FI <0.2 (that is, with fewer than nine of 42 deficits) develop dementia over 10 years (Figure 3).

**Table 3:** Odds ratios for death and dementia using the all-factor frailty index (age adjusted)

**Outcome**	**B**	**SEM**	**Wald**	**Exp(B)**	**95% CI**	
					**Lower**	**Upper**
Death (Men)						
Age	0.18	.011	285.73	1.20	1.17	1.22
Frailty Index	0.20	.018	125.36	1.22	1.18	1.26
Constant	−13.63	.776	308.84	0.00		
Death (Women)						
Age	0.17	.008	442.26	1.19	1.17	1.21
Frailty Index	0.13	.011	131.79	1.14	1.11	1.16
Constant	−13.63	.604	509.15	0.00		
Dementia (Men)						
Age	0.19	.017	118.34	1.20	1.17	1.25
Frailty Index	0.17	.028	35.92	1.18	1.12	1.25
Constant	−15.63	1.259	154.14	0.00		
Dementia (Women)						
Age	0.22	.013	281.86	1.25	1.22	1.28
Frailty Index	0.07	.016	19.31	1.08	1.04	1.11
Constant	−18.43	.999	340.62	0.00		

Note: Values of the index are presented by using the deficit count (for example, we multiplied the index by 42 for the all-factor measure), to evaluate the change in risk seen with each added deficit. As a worked example, for each increase in deficit accumulation, the risk of death increased by 22% in men, 14% in women; for dementia, for each increase in deficit accumulation, the risk of dementia increased by 18% in men and 8% in women for dementia. Significance level, *P* < 0.001 for age and Frailty Index in each model.

Regarding objective 2, in a multivariable logistic regression model adjusted for age, when the number of deficits considered in the index increased, the ORs for death and dementia each increased for the index. Likewise, the impact of age decreased. For death, when the number of deficits included in the index increased from 5 to 42, the Wald statistics decreased by 16% for age (from 341to 286) and increased by 71% for the index in men (from 37 to 126). For women, the corresponding changes were a 22% decrease for age and a 73% increase for the FI. For dementia, when the number of deficits included in the index increased from 5 to 42, the values of the Wald statistic were a 15% decrease for age and 64% increase for the index in men; 11% decrease for age and 63% increase for the index in women. By contrast, the change in the beta estimates for the constants were negligible: -13.63 versus -13.72.

**Figure 4:**
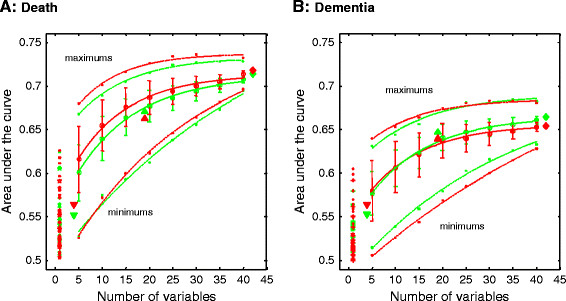
**C-statistics (areas under the curve) of the indices in the prediction of death (left panel) and dementia (right panel) as a function of the number of deficits considered in the indices, in men (green) and in women (red).** Symbols represent the observational data, and lines represent the curve fitting to a reversed exponential function (R^2^ > 0.960; *P* < 0.001). Data were obtained by using randomly selected variables with 1,000 simulations. Solid symbols on the left show the performance with use of each individual deficit (traditional risk factors, stars; nontraditional, asterisks; others, dots). Performance of the nontraditional, traditional, and all risk-factor indices are shown by the larger down-triangles, up-triangles, and diamond symbols.

The FI, constructed by using all 42 variables, best predicted subjects who died and those who developed dementia over 10 years (Figure 4: diamond symbols; AUC = 0.71 ± 0.02 for men, 0.72 ± 0.02 for women for death; AUC = 0.67 ± 0.03 for men, 0.66 ± 0.03 for women for dementia). The predictive power was associated with the total number of variables considered: by using any given variable, the AUC for dementia was usually <0.55, and seldom exceeded 0.60. The AUC increased with the number of randomly selected variables under consideration, and gradually stabilized when *n* > 25, at which the values converged (Figure 4).

## 4 Discussion

In this secondary analysis of a well-established population-based study of cognitive health, we examined the predictive power of risk indices constructed by using differing types and numbers of deficit variables for 10-year dementia and mortality. The data showed that the death and dementia outcomes were both closely associated with baseline health status, as represented by the level of deficit accumulation in an FI. More interesting, the predictive value of the risk indices increased as a function of the number of deficits included in the index. Here, the deficits covered a wide range of self-reported health problems, including diseases, symptoms, and disabilities, some of which were known dementia risk factors, and some were not. As such, our study provides evidence suggesting that the risk of dementia and death may be determined more by the overall state of health, and notably more by how many health problems an individual has, and less by exactly what these problems are. This general result held true for both men and women.

In other words, each deficit adds information, even those that individually do not significantly increase dementia risk. It was also interesting to note that for both death and dementia, women tolerated deficits better than men did, as evidenced by lower risks per deficit. This result parallels similar investigation by our group and others in understanding age-related deficit accumulation and the risk of death [22],[23].

It is well understood that people are more likely to develop health problems at variable rates as they age. Important for our study, the aging brain subject to late-life cognitive dysfunction appears to be especially deficit prone [16]. For example, just over the past decade, more than 20 dementia risk factors have been identified for sporadic Alzheimer disease, the most common cause of late-life dementia, which are in addition to advanced age and genetic determinants (for example, carrying the ApoE *e*4 allele). This long list has included, in addition to the items cited at the outset from 2014 [1]-[4], traumatic head injury, stroke and transient ischemic attack (TIA), hypertension, heart diseases, diabetes mellitus, obesity, high-fat diet, metabolic syndrome, low dietary intake of antioxidants, fish, vegetables, or fruits, reduced high-density lipoprotein cholesterol, deficiency of vitamin B or trace minerals, hypothyroidism, high homocysteine, abnormal serum-hemoglobin level, sleep apnea, anxiety, depression, poor resilience, anemia, smoking, alcoholism, anesthesia, environmental toxic exposure and pollution, low physical, cognitive, or social activities, and low education, income, or social status [10],[23]-[27].

Although it is reasonable to expect that more health problems will join this long list, our study supports aggregating these items to assay whether a new “risk factor” is informative. In short, we support the proposal that this is best done explicitly, in a frailty/deficit accumulation index, rather than implicitly, through age [9]. Here too, we note that age is not part of the FI. Instead, the FI represents an alternative way to tease out information that otherwise might be lost in age-adjusted models. That consideration, too, informed the strategy of evaluating the impact of age in the multivariable logistic regression model as we increased the number of health deficits that we included in the FI (Figure 4): the decline in the effect of age is another way of demonstrating the cumulative increase in information for the FI as more items are included in it.

In this way, our study also contributes to the current literature relating general health to adverse outcomes. An FI composed of a variety of health deficits has been used to predict multidirectional changes in cognitive test scores (for example, getting worse, getting better, or remaining the same), and not just in relation to dementia/no dementia, or a dichotomous outcome that includes the “cognitive impairment, not dementia” category [28],[29]. An index made up of baseline health deficits that are neither cognitive risk factors (for example, hypertension, heart disease), nor cognition-related measures (for example, foot problems) has also been found to predict dementia [18]. More recent work has extended this approach from dementia to other multiply determined illnesses that are both common and age associated.

In a re-analysis of data from the Canadian Heart Health Survey, non-traditional risk factors were combined to predict an elevated risk of coronary heart disease, and remained so in analyses adjusted for age and for traditional risk factors, also combined in a risk-factor index [30]. In short, consider the analogy with the presentation of late-life disease. When many other illnesses are present, it is more likely to result in so-called “atypical” manifestations; for example, myocardial infarction in a frail patient is as likely to be seen with delirium or a fall, whereas older adults with heart disease and not a lot of other health deficits are more likely to have chest pain [8],[31]; so too must we accept that in people with many health deficits, it is unwise not to consider their combined effect.

Can this approach of aggregating risk factors aid our understanding of mechanisms? The FI approach has been criticized for lacking such specificity [16], but it might in fact be more revealing in exactly this way. Consider that many of the risk factors included in the FI would appear to have little in common with each other, except that they are injurious in some way. This is true of a larger set of risk factors that might include other items not measured here, such as paternal age at birth, or specific toxin exposures, or details about head injury. So at a broad level, if what these risk factors have in common is the need for the body to mount responses, then in people with aberrant repair processes, or exhausted ones, various types of damage can accumulate [5]. This can be formulated precisely, by using the mathematics of queuing theory. In brief, deficit accumulation occurs when the number of insults to which an organism is exposed (that is, the damage rate) surpasses the ability for the damage to be either removed or repaired, and so deficits occur and accumulate [32]. The length of a queue (here, the number of deficits) is a function of the number of people arriving at the queue (for us, the damage rate), and how quickly the people can be processed in the queue (here, the repair/removal response). Indeed, a recent study of the neuropathology of Alzheimer disease in relation to frailty showed the best explanatory power only when Alzheimer disease pathology, macroinfarcts, and nigral neuronal loss all were considered in a single model [33].

In short, if we accept that cognition in older adults is formally complex and dynamic (and this can be demonstrated with simple clinical and imaging tools [29]), then it will be important, in achieving a fuller understanding of how cognition becomes impaired, to have tools that allow dynamic changes to be understood. That this approach is indifferent to exactly which mechanisms are involved is more a strength than a weakness. Second, from a clinical standpoint, this is a cautionary note to take into account the general health of the patient, a focus that can get lost in subspecialty practice. Our data suggest that good management of people with dementia should pay attention to other active illnesses as well as medical management.

Our data must be interpreted with caution. The results underscore the competing outcome effect between death and dementia: subjects might die before they have a chance of developing dementia. Here, subjects who had troubles with kidney, bladder control, chest pain, high blood pressure, heart/circulation at baseline were at such a high risk of death that these factors did not increase the risk of dementia in the survivors. Moreover, whereas 10-year survival status was known for all participants, cognitive reassessments were made at the 5-year follow-ups in CSHA. In consequence, some subjects could have died with undiagnosed dementia. This, and the 14% survivors with an unknown cognitive outcome, made it possible that the dementia rate of the sample was somewhat underestimated. Note too, in the index variables, we treated each deficit equally (that is*,* without variable weighting), even though it is clear that different health problems could contribute differently to an outcome, and the predictive value may appear higher with use of weighted scales [9],[11],[34]. An obvious advantage of not assigning a specific weight to a variable is that it improves generalizability, as the result may depend less on which exact deficits are available to be used.

Our data contribute to the discussion about biomarkers. Whatever their specific advantage, even individually significant measures such as early amyloid beta retention, CSF, serum, and neuroimaging biomarkers generally result in no better than moderate prediction accuracy [17],[35],[36]. This may simply reflect that too few biomarkers are being deployed to account for the various ways in which dementia can develop. Further, whatever the statistical technique used, it seems likely now that, from a biologic standpoint, the independence of one biomarker from another must be reexamined. In this regard, the parallel case exists in relation to mortality prediction in relation to health deficits, in which different subsets of variables can give comparable results in the prediction of adverse outcomes; many commentators note that this reflects not just diversity in individual health, but multiple dependencies among health measures [8],[16]. In that case too, whether a set of variables represents “the best” predictors varies by target outcomes, even within the same dataset. Here, the predictive value of the index became increasingly greater as the number of variables used increased, until it was sufficiently large (*for example,* >25). Whether further information offsets noise in the data requires further evaluation in other datasets. Given that a complex system can be reflected by the redundancy of acquired deficits [8],[19], when a sufficient number of deficits are taken into account, each adding a bit of information to the system’s profile, resulting in broad coverage of diverse health states, the selection of a small number of specific variables shows no advantage.

Here, too, we observed a relatively low predictive value for dementia, which was lower than death prediction. One possibility is that dementia represents a state that can involve complex origins related to the dynamics of brain structure and function [29],[37]. In addition, relations of risk factors to dementia outcomes are complicated, varying by follow-up duration and sample profiles [27],[38],[39]. In this regard, evaluations of structural and functional brain health can be useful [27],[40],[41], enlightened partially by studying whole-brain atrophy and lesion changes in aging and dementia [29],[42]. Further research will test how health status combined with brain changes can help with dementia prediction.

## 5 Conclusions

The predictive value of 10-year dementia risk varies by the number of deficits considered. Traditional and nontraditional risk factors did not have significantly different predictive ability. The variety of items associated with dementias suggests that some part of the risk might relate more to aberrant repair processes than to specifically toxic insults. The epidemiology of late-life illness must consider overall health status.

## Abbreviations

ApoE4: Apolipoprotein E4 allele

AUC: area under the curve

CI: confidence interval

CSF: cerebrospinal fluid

DSM-III-R (and DSM-IV): Diagnostic and Statistical Manual of Mental Disorders, third edition,rsion- revised (and DSM, fourth edition)

OR: odds ratio

ROC: receiver operating characteristic

## Competing interests

No authors have any financial competing interests related to this work. XS and AM receive research funding from the Canadian Institutes of Health Research on secondary data-analysis projects and have no conflict of interest with this work. KR receives research grants from the Canadian Institutes of Health Research, funding from Dalhousie Medical Research Foundation as Kathryn Allen Weldon Professor of Alzheimer Research, and a fellowship from the Alzheimer Society of Canada, and has the following financial disclosures. Journal advisory board member: *Neuroepidemiology* (2002-), *Journal of Gerontology, Medical Sciences* (2003-), *Alzheimer’s Research and Therapy* (2008-), *BMC Medicine* (2009-), and *Chinese Journal of Geriatrics* (2010-). Employment: President & Chief Scientific Officer: DementiaGuide, Inc.; Stocks: DementiaGuide, Inc. Legal Proceedings: Expert witness for Tory’s LLP, on behalf of Eisai and Pfizer Canada.

## Authors’ contributions

XS processed and analyzed the data, prepared the result presentation, and drafted various versions of the manuscript. AM helped with data analysis and result presentation, verified the analysis outcomes, and helped draft the manuscript. KR conceived the research concept, secured funding for conducting the secondary analysis, verified the analysis outcomes, and helped draft the manuscript. All authors edited and approved the final version of the manuscript.
